# Ambient aromatic hydrocarbons and prostate cancer: mechanistic evidence linking benzene and PAH exposure to tumor progression

**DOI:** 10.3389/fcell.2026.1836387

**Published:** 2026-07-29

**Authors:** Hao Wu, Hongliang Cao, Zihao Ye, Zhanhao Li, Mingyang Chang, Wenqiang Zhang, Tao Xu, Lei Wang, Tong Yang, Xilin Deng, Enrui Hu, Baoshan Gao

**Affiliations:** Department of Urology, First Hospital of Jilin University, Changchun, Jilin, China

**Keywords:** AhR–AR crosstalk, aromatic hydrocarbons, immune remodeling, oxidative stress, polycyclic aromatic hydrocarbons, prostate cancer

## Abstract

Prostate cancer (PCa) remains one of the most common malignancies in men worldwide, yet modifiable environmental contributors remain incompletely defined. Aromatic hydrocarbons (AHs), particularly benzene and polycyclic aromatic hydrocarbons (PAHs), are widespread pollutants in ambient air, occupational settings, tobacco smoke, and high-temperature cooking emissions. In this review, we synthesize evidence from population-based and occupational epidemiology, dietary exposure proxies, animal carcinogenesis models, organoid systems, and cell-based mechanistic studies to assess the biological plausibility of AH-related prostate carcinogenesis. Human studies support modest but recurrent associations between long-term exposure to traffic-related mixtures, benzene-containing emissions, PAH-generating cooking practices, and increased PCa risk, with stronger duration–response signals in occupational settings. Experimental evidence further indicates that benzo[a]pyrene and related PAHs can induce prostatic mutagenesis, oxidative and genotoxic stress, endocrine perturbation, epigenetic remodeling, and immunosuppressive changes in the tumor microenvironment. The most coherent mechanisms involve AhR–AR crosstalk, CYP1A1/1B1-mediated bioactivation, ROS generation, DNA-adduct formation, DNMT1/HDAC6-associated epigenetic regulation, JAK2/STAT3-linked survival signaling, epithelial–mesenchymal transition, and IDO/TDO–kynurenine–AhR-mediated immune suppression. Collectively, current evidence supports a biologically plausible framework in which AH exposure may contribute to PCa initiation and progression and may intersect with pathways implicated in therapy resistance, although causal inference remains constrained by exposure misclassification, mixture complexity, and limited longitudinal biomarker data. Future studies should integrate precise exposure assessment with prostate-specific molecular studies, organoid systems, animal models, and prospective human cohorts.

## 1 Introduction

Prostate cancer (PCa) is a heterogeneous epithelial malignancy with a highly variable clinical course. Some tumors remain indolent and organ-confined for years, whereas others progress rapidly, metastasize early, and eventually become resistant to androgen-directed therapy. Over recent decades, the global incidence and prevalence of PCa have continued to rise, making it one of the most commonly diagnosed cancers in men (Bray et al., 2024; James et al., 2024). Although advances in surgery, radiotherapy, androgen-deprivation therapy, and targeted systemic treatments have improved outcomes, several unresolved problems remain. Screening still lacks optimal specificity and sensitivity, allowing underdiagnosis and overtreatment to coexist (Bray et al., 2024; Fazekas et al., 2024). Management of advanced disease is also limited by treatment-related toxicity, biological heterogeneity, and variable therapeutic responses (Khorram et al., 2024). Preventive strategies remain comparatively underdeveloped, partly because modifiable environmental contributors to prostate carcinogenesis are still incompletely characterized. Progress in this area will therefore require a clearer understanding of how environmental exposures act at the levels of molecular initiating events, cellular stress responses, and tumor-microenvironment remodeling.

Aromatic hydrocarbons (AHs), a major subclass of volatile organic compounds (VOCs), are ubiquitous pollutants encountered in outdoor air, indoor environments, food, and many occupational settings (Zhou et al., 2023). AHs include monocyclic aromatic hydrocarbons such as benzene, toluene, ethylbenzene, and xylene, as well as polycyclic aromatic hydrocarbons (PAHs) such as naphthalene and benzo[a]pyrene. Major exposure sources include traffic and industrial emissions, tobacco smoke, solvent-containing products, high-temperature cooking, contaminated food or water, and occupational contact with combustion- or petroleum-derived mixtures (Sampaio et al., 2021; Kamani et al., 2023; Kumari et al., 2024). Because many AHs are semi-volatile and lipophilic, they can enter the body through inhalation and ingestion, circulate systemically, and interact with xenobiotic-metabolizing and steroid-related pathways that are relevant to prostate biology. Epidemiological and occupational studies have reported associations between traffic-related VOCs, benzene, PAH-rich mixtures, and PCa risk, while experimental studies suggest that selected AHs may contribute to prostatic tumor initiation and progression through genotoxic, endocrine, inflammatory, and microenvironmental mechanisms (Goldberg et al., 2022). In this review, we focus primarily on the cellular and molecular mechanisms linking AH exposure to PCa biology. Epidemiological, occupational, dietary, animal, organoid, and cell-based evidence is used to establish exposure relevance, biological plausibility, and evidence hierarchy, rather than to frame the article as a broad epidemiological or public-health review. The central mechanistic framework is organized around AhR-mediated xenobiotic sensing, CYP1A1/1B1-dependent bioactivation, KEAP1–NRF2-regulated oxidative stress responses, DNA-adduct formation and genotoxic injury, androgen receptor crosstalk, epigenetic remodeling, potential vascular and stromal remodeling, and inflammatory and immune remodeling of the tumor microenvironment. By separating association-level evidence from experimental and molecular evidence, this review aims to clarify how AHs may contribute to PCa initiation, progression, and therapy resistance, while also identifying key mechanistic gaps for future research.

## 2 Evidence landscape and biological plausibility across study systems

Evidence linking AH exposure to PCa spans several biological and methodological levels, including population-based studies, occupational exposure analyses, dietary exposure proxies, human biomarker and tissue studies, cell and organoid models, and *in vivo* carcinogenesis experiments. These lines of evidence do not carry the same inferential weight. Population and occupational studies establish exposure–disease associations and dose–response patterns, but remain vulnerable to exposure misclassification, screening differences, and residual confounding (Barul and Parent, 2021; Goldberg et al., 2022; Chen Y. et al., 2025). By contrast, experimental models provide stronger causal and mechanistic support by linking selected AHs, particularly PAHs such as BaP and DMBA, to DNA damage, oxidative stress, endocrine perturbation, immune remodeling, and tumor progression (Krais et al., 2016; Shi et al., 2017; Xue et al., 2018; Gao et al., 2020). Therefore, this section is organized as an evidence landscape rather than as a stand-alone epidemiological review. Its purpose is to define exposure relevance, identify biological plausibility, and distinguish association-level evidence from experimental and molecular evidence before the mechanistic pathways are examined in Section 3.

### 2.1 Human population and dietary exposure evidence: association signals and exposure proxies

At the human population level, traffic-related aromatic compounds, particularly benzene within VOC mixtures, have been associated with PCa risk (Li J. et al., 2024). Complementary network toxicology and machine-learning analyses have prioritized pollutant-responsive PCa-related hubs, including HDAC6, CDK1, DNMT1, NOS3, and DPP4. These computational findings are treated here as hypothesis-generating mechanistic signals rather than as direct population-level evidence (Li Y. et al., 2025; Liu et al., 2025). Among exposure-measured epidemiological studies, the Montreal *PROtEuS* case–control study (1,172 cases/1,177 controls) used high-resolution exposure surfaces for five VOCs and revealed monotonic, non-linear exposure–response functions for residential benzene and ethylbenzene; moving from the 5th to the 25th percentile of benzene exposure corresponded to approximately two-fold higher odds of incident PCa, even after adjusting for screening and tumor grade (adjusted OR ≈ 2.00, 95% CI 1.47–2.71) (Goldberg et al., 2022). Complementing these findings, a UK Biobank cohort of 210,722 men reported positive associations between PCa risk and interquartile-range increases in PM2.5, PM10, NO2/NOx, and benzene. Multi-pollutant models remained positive, and neighborhood greenness attenuated risk estimates, consistent with a traffic-related mixture signal in which AHs such as benzene may represent one biologically relevant component (Chen Y. et al., 2025). However, proximity-based evidence is less consistent. In MCC-Spain, living near industrial installations was not associated with an overall excess risk of PCa, although selected industrial sectors and specific emissions showed positive associations. The inverse associations observed for some emissions categories, including PAHs, highlight the limitations of coarse exposure proxies and the potential influence of land use, socioeconomic factors, and exposure misclassification (García-Pérez et al., 2024).

Dietary studies provide additional exposure proxies rather than direct mechanistic proof. High-temperature cooking and greater doneness can increase PAH and HCA formation, and studies of well-done meat, deep-fried foods, and gene–environment interactions have reported associations with PCa risk or aggressive disease phenotypes (Koutros et al., 2008; Stott-Miller et al., 2013; Iwasaki and Tsugane, 2021; Oczkowski et al., 2021; Reng et al., 2022). These studies support the idea that cooking-related mutagen formation, rather than diet category alone, may be the more relevant exposure construct. Gene–environment data further suggest that host susceptibility can modify these diet–exposure relationships; for example, variation in inflammatory or xenobiotic-response pathways may influence the association between high-temperature–cooked foods and advanced PCa (Asadian et al., 2021). By contrast, meta-analyses of total red-meat intake *per se* show little or no association with overall or advanced PCa, suggesting that preparation-related mutagen formation may be more relevant than meat quantity itself (Nouri-Majd et al., 2022).

Overall, human population-based evidence supports an association between long-term exposure to traffic-related mixtures, benzene, and cooking-related PAH/HCA proxies and PCa risk, but the strength and specificity of these associations vary across exposure settings (Goldberg et al., 2022; Chen Y. et al., 2025; Nouri-Majd et al., 2022). This interpretation is consistent with broader evidence linking air-pollution mixtures to urological cancer risk, although such studies often capture mixed pollutants rather than AH-specific exposure (Li J. et al., 2024). Heterogeneity across studies likely reflects exposure misclassification, differences in screening practices, broad air-pollution surrogates, and residual social or behavioral confounding. Within population-based and dietary evidence, the signal appears more interpretable when exposure is estimated with greater chemical specificity, such as benzene or cooking-related PAH/HCA proxies, and less stable when exposure is inferred from residential proximity or broad environmental indicators. This gradient suggests that exposure intensity, duration, chemical specificity, and measurement quality substantially influence effect estimates. Importantly, these human data should be interpreted as association-level evidence. Their primary value in this review is to justify mechanistic interrogation of AhR–AR crosstalk, CYP1A1/1B1-mediated bioactivation, oxidative stress, inflammatory signaling, and susceptibility-related pathways (Gao et al., 2020).

### 2.2 Occupational exposure evidence: cumulative exposure gradients and susceptibility

Occupational studies are important in this evidence landscape because workplaces often involve higher-intensity, longer-duration, and better-characterized AH mixtures than general environmental settings, making exposure–response gradients easier to evaluate (Barul and Parent, 2021; Lee et al., 2023; Barul et al., 2025). Relevant occupational scenarios include petrochemical work, automobile manufacturing, printing, firefighting, and other combustion- or solvent-related activities, in which workers may encounter benzene, toluene, soot, PAHs, and mixed hydrocarbon by-products (Seidler et al., 1998; Kim et al., 2023; Lee et al., 2023). Across hydrocarbon-rich workplaces, the clearest signals generally arise in settings with long-duration or substantial exposure to mixed AHs (Lee et al., 2023). In Montreal’s *PROtEuS* occupational analysis, expert-rated lifetime work histories indicated that ever-exposure to monocyclic AHs, including BTX and styrene, was associated with higher overall PCa risk (OR 1.27, 95% CI 1.05–1.53) (Blanc-Lapierre et al., 2018). This association was more apparent for low-grade tumors and showed a duration–response pattern for “any BTX”; exposure for ≥25 years at substantial levels yielded ORs of approximately 2.3–2.4 for low-grade disease, independent of screening (Barul and Parent, 2021; Barul et al., 2025). Additional studies using industry- or process-based proxies also suggest modest excess risks in hydrocarbon-rich settings. For example, a large nested case–control study in motor-vehicle manufacturing observed higher odds for casting operations, including core/mold making (OR 1.5) and metal melting/pouring (OR 1.9), which are processes characterized by PAH-rich mixtures. However, estimates varied across facilities (Calvert et al., 1998; Brown and Delzell, 2000). Source-specific PAH analyses further illustrate both the value and the difficulty of occupational exposure reconstruction. When lifetime PAH exposure in *PROtEuS* was reconstructed at the job level and parsed by likely source, overall associations attenuated after lagging and restriction to probable or definite exposure. Nevertheless, small excesses were observed for wood-derived PAHs, and higher point estimates were reported for high-grade tumors. Similar patterns have been described among firefighters, while analyses addressing screening behavior suggest that differential screening alone is unlikely to fully explain these associations (Barul and Parent, 2021; Lee et al., 2023; Marjerrison et al., 2025). These occupational findings provide a higher-exposure human context for mechanism-oriented interpretation, while mixed exposures, retrospective job-exposure reconstruction, and exposure-source heterogeneity remain important limitations.

Occupational PAH exposure may be translated into molecular injury through PAH–DNA adduct formation and oxidative stress in prostate-relevant tissues, and inter-individual differences in detoxification capacity, including GSTP1-related pathways, may modify susceptibility (Dasgupta et al., 2020; Santric et al., 2021; Kawahata et al., 2022; Ye et al., 2023). In Detroit, a case-only gene–environment analysis found over-representation of GSTP1 Val105 carriers among cases in the highest quartile of petroleum-related respiratory PAH exposure (OR 1.74–1.85 overall; OR 4.52 in men younger than 60 years), consistent with susceptibility-dependent risk under high exposure (Santric et al., 2021; Ye et al., 2023). Evidence from complex exposure settings should be interpreted more cautiously. A 2025 systematic review and meta-analysis in U.S. veterans reported a statistically significant but modest excess risk associated with hydrocarbon exposure (pooled OR 1.14, 95% CI 1.01–1.28). Because these settings involve mixed exposures rather than isolated AHs, they are best viewed as supportive exposure-context evidence rather than chemical-specific proof (Green-Lott et al., 2025). Altogether, occupational evidence supports the relevance of long-term or cumulative AH exposure and susceptibility-related modifiers, while also underscoring the limitations of mixed exposures and retrospective exposure assessment. These observations provide a human high-exposure bridge to the mechanistic pathways discussed in Section 3, particularly CYP1A1/1B1-dependent bioactivation, oxidative and inflammatory imprinting, DNA-adduct formation, and immune microenvironmental remodeling.

### 2.3 Experimental model evidence: initiation, progression, and mechanistic support

Experimental models provide more direct support for the causal and mechanistic involvement of selected AHs, particularly PAHs, in prostate carcinogenesis and tumor progression. Experimental evidence from mouse mutagenesis models links BaP exposure to prostatic genetic injury; complementary PAH–DNA adduct evidence in prostate-relevant tissues further supports the plausibility of PAH-driven initiation within the gland (Guttenplan et al., 2001; Nascimento-Goncalves et al., 2023; Naiki-Ito et al., 2025; Zhang Z. et al., 2025). Rat models using androgen-promoted carcinogenesis further demonstrate that PAH exposure can increase tumor incidence or tumor burden under permissive hormonal conditions (Ibrahim et al., 2022; Bakam et al., 2023; Nascimento-Goncalves et al., 2023). In a Wistar protocol using testosterone priming followed by an intraprostatic BaP challenge, tumor incidence reached 75% in exposed animals (Bakam et al., 2023). Notably, chemopreventive treatment with Cucumis sativus seed oil sharply attenuated this signal, reducing incidence to 12.5%, normalizing PSA and inflammatory cytokines, and restoring antioxidant defenses—readouts that together underscore BaP’s tumor-promoting and oxidative/inflammatory imprint *in vivo* (Bakam et al., 2023). PAH-driven carcinogenesis is likewise reproduced with 7,12-dimethylbenz[a]anthracene (DMBA) under androgenic promotion (Nascimento-Goncalves et al., 2023; Ibrahim et al., 2022). In a testosterone–DMBA model, a marine Bacillus-derived acidic exopolysaccharide (EBPS) substantially reduced PCa burden: EBPS lowered PSA, suppressed 5α-reductase (5α-R) and Na^+^/K^+^-ATPase activities (enzymes tied to growth signaling and cellular energetics), improved survival, and decreased tumor mass—illustrating that a PAH-initiated process can be tempered by interrupting oxidative/inflammatory and hormonal co-drivers *in vivo* (Ibrahim et al., 2022).

Beyond initiation, BaP accelerates progression and reshapes host defenses. In a mouse xenograft system, systemic BaP exposure enhanced tumor growth while diminishing intratumoral CD4^+^ and CD8^+^ T-cell infiltration, indicating an immunosuppressive microenvironment. Patient-derived organoids similarly proliferated faster under BaP, and transcriptomic profiling implicated apoptosis-regulatory and androgen-signaling nodes as candidate mediators (Zhang Z. et al., 2025). Mechanistic animal data further link PAH bioactivation in prostate tissue to heightened carcinogenic potency (Maksymchuk et al., 2024; Mokhosoev et al., 2024). In rats, vitamin E, although generally considered an antioxidant, increased prostatic CYP1A1/1B1 and related xenobiotic-activating enzymes, raised ROS with lipid/protein oxidative injury, and amplified BaP-driven cell transformation *in vitro* and *in vivo*, consistent with a co-carcinogenic effect mediated by enhanced PAH bioactivation within the prostate milieu (Jiang, 2024). Together with evidence that the prostate expresses the enzymatic machinery required for BaP activation and DNA-adduct formation, these observations help explain why PAH exposures can be both initiating and promoting in animal prostates (Maksymchuk et al., 2024; Caipa Garcia et al., 2024; Bukowska et al., 2022). Across species and paradigms—chronic BaP exposure in mice; BaP- or DMBA-initiated rat carcinogenesis with androgenic co-promotion; and BaP-exposed tumor-bearing mice—PAHs reproducibly increase risk or hasten progression, with effects partially reversible by antioxidant/anti-inflammatory or hormonal-axis interventions (Ibrahim et al., 2022; Bakam et al., 2023; Naiki-Ito et al., 2025). Together, these experimental models show that PAH exposure can contribute to prostate tumor initiation, progression, oxidative–genotoxic injury, endocrine crosstalk, and immune remodeling under defined experimental conditions (Dai et al., 2023; Zhang Z. et al., 2025). These findings provide causal and mechanistic support that complements the more modest and potentially confounded associations observed in human population and occupational studies, and they set up the pathway-focused discussion in Section 3.

## 3 AHs influence PCa through multiple intersecting biological pathways

**FIGURE 1:**
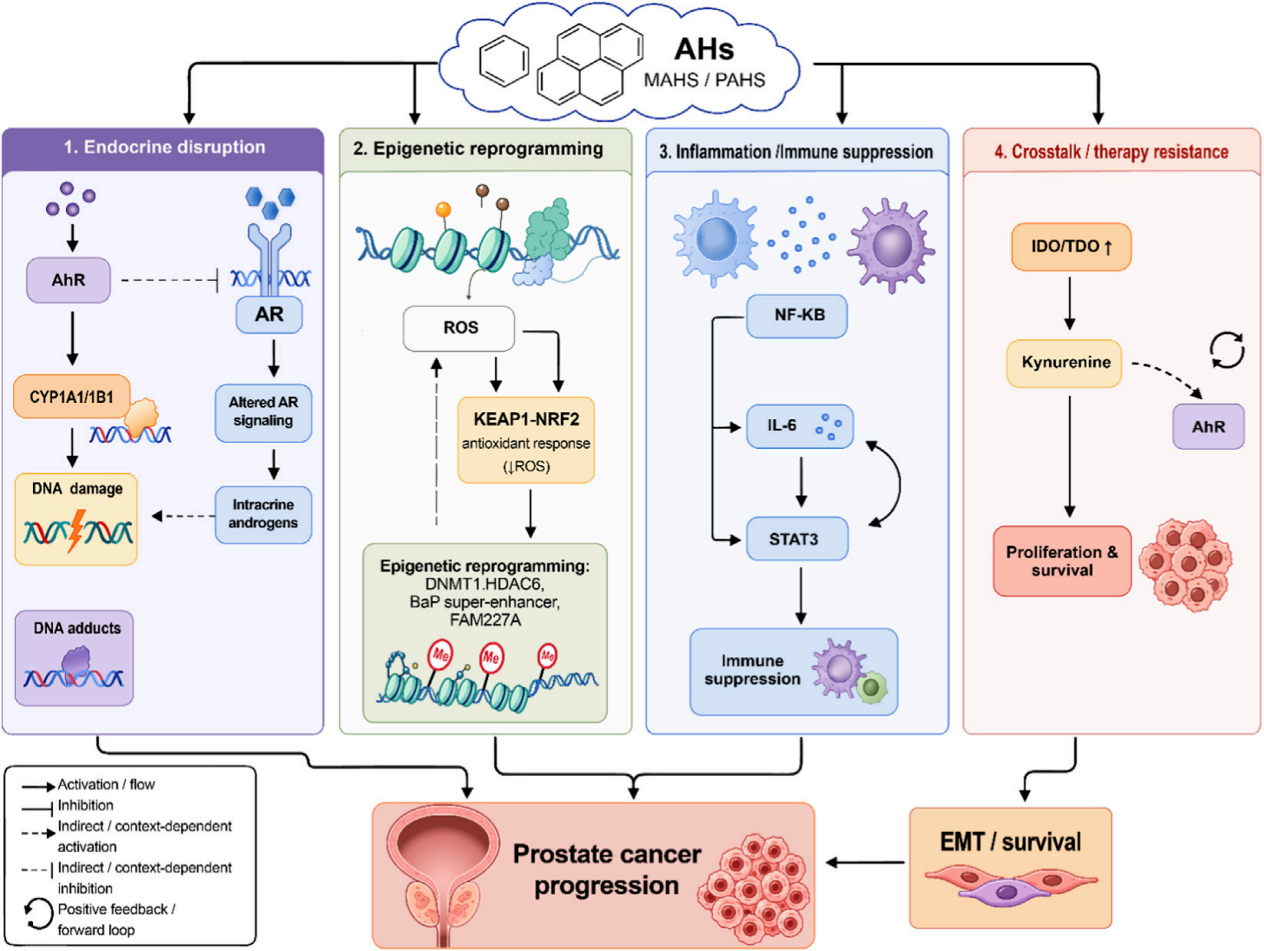
Integrated model of aromatic hydrocarbon–driven prostate cancer progression. AHs engage AhR-centered signaling and intersect with AR regulation, CYP1A1/1B1-mediated bioactivation, ROS generation, DNA-adduct formation, KEAP1–NRF2 antioxidant responses, DNMT1/HDAC6-associated epigenetic reprogramming, NF-κB–IL-6–STAT3 inflammatory signaling, and IDO/TDO–kynurenine–AhR immune-suppressive crosstalk. These pathways converge on endocrine disruption, immune suppression, proliferation/survival, EMT, and prostate cancer progression. Solid arrows indicate activation or flow; T-bars indicate inhibition; dashed lines indicate indirect or context-dependent links; ↻ indicates feedback or feed-forward loops. “Me” denotes methylation marks, and “P” denotes phosphorylation.

Current evidence suggests that environmental AHs may influence the development and progression of PCa, with converging findings from *in vitro* systems, *in vivo* models, and population studies supporting a multi-level mechanistic framework. Taken together, AHs appear to contribute to prostate carcinogenesis by disrupting endocrine signaling, provoking oxidative stress and DNA damage, reprogramming the epigenome, disturbing the proliferation–apoptosis balance (including EMT), and remodeling inflammatory and immune responses—processes that operate from initiation through progression and, in some cases, persist beyond the exposure window. After exposure, AHs engage sensor receptors, most prominently AhR, and trigger downstream cascades that promote hormonal dysregulation, genomic instability, and a tumor-promoting microenvironment, while oxidative-stress response pathways, notably the KEAP1–NRF2 axis, modulate cellular adaptation to persistent redox stress (Tossetta et al., 2023a; Szaefer et al., 2024). These pathways seldom operate in isolation; instead, they interact across stages of PCa initiation, promotion, and progression through feedback and feed-forward loops involving AhR, AR, CYP1 enzymes, ROS, NF-κB, JAK2/STAT3, and inflammatory mediators (Singh et al., 2024; Szaefer et al., 2024). From this perspective, endocrine disturbance may heighten susceptibility to genotoxic injury, whereas chronic inflammation can compound oxidative stress and tune epigenetic control, thereby priming chromatin for durable transcriptional shifts that may outlast the original exposure (Wang et al., 2023; Szaefer et al., 2024; Zhang L. et al., 2025). To clarify levels of biological organization, we distinguish direct mechanistic evidence from prostate epithelial cells, tumor-cell lines, and organoid models; supportive causal evidence from *in vivo* and xenograft systems; and contextual evidence from human biomarker, occupational, and population studies. Mechanistic inference is therefore weighted most strongly toward cellular and animal data, whereas human observational findings are treated as external support for biological plausibility (Table 1). Figure 1 summarizes the major mechanisms through which AH exposure may contribute to prostate carcinogenesis, highlighting key genes, signaling pathways, and therapy-resistance-related crosstalk.

**TABLE 1:** Evidence linking aromatic hydrocarbon exposure to PCa across study types.

Evidence type	Country/region and cohort/design	Exposure source/class	Study (authors, year)	Exposure–response	Results
Population-based studies	Montreal, Canada; population-based case–control (*PROtEuS*)	Ambient VOCs (benzene, ethylbenzene, etc.)	Goldberg et al. (2022)	Monotonic, non-linear functions; ∼2× higher odds from low to mid percentile of benzene	Higher odds of incident PCa with increasing benzene/ethylbenzene; robust across grades
	Prospective cohort	Multi-pollutant ambient mix (PM2.5/PM10, NO2/NOx, benzene)	Chen Y. et al. (2025)	Linear trends; multi-pollutant models remained positive	Positive associations per IQR for PM/NOx and benzene; joint effects significant
	United States; Agricultural Health Study cohort	Dietary HCAs/PAHs from high-temperature meat	Koutros et al. (2008)	Dose–response across doneness/HCA load	Elevated incident PCa and ∼2× higher advanced PCa with well/very-well-done meat
	United States; population-based case–control (Seattle)	Deep-fried food (PAHs/aldehydes/HCAs)	Stott-Miller et al. (2013)	Category trend across intake frequency	Higher PCa odds with ≥weekly fried foods; stronger for aggressive tumors
	Spain; MCC-Spain multicase–control	Proximity to industrial installations (mixture incl. AHs)	García-Pérez et al. (2024)	Heterogeneous; some positive proximity gradients	Overall null; sector-specific elevations; pollutant-specific positive signals
Occupational exposure studies	Canada; population-based case–control	BTX/styrene	Blanc-Lapierre et al. (2018)	Duration/level gradients for BTX	Modest ↑ risk overall; signals more evident for low-grade disease
	Canada; population-based case–control	Occupational PAHs	Barul and Parent (2021)	Duration–response in select subgroups	Small ↑ risk; patterns consistent with firefighter/combustion exposures
	United States; nested case–control in motor-vehicle mfg.	Combustion/PAH-rich operations	Brown and Delzell (2000)	Job/time in high-exposure operations	Higher odds in casting core/mold and melt/pour operations
	Germany; case–control	Diesel exhaust (PAH/BTX mixture)	Seidler et al. (1998)	Exposure intensity and duration trends	Elevated PCa odds in diesel-exposed jobs
Animal model evidence	MutaMouse; *in vivo* mutagenesis	Oral BaP; dietary lycopene modulation	Guttenplan et al. (2001)	Clear dose/agent effect; dietary attenuation	BaP induced prostate mutagenesis; lycopene diet reduced BaP-induced mutations
	Wistar rat; BaP + androgen promotion	Intraprostatic BaP + testosterone; seed-oil intervention	Bakam et al. (2023)	Exposure–response in incidence; intervention reversed risk	Prostate tumor incidence ∼75% with BaP; reduced to ∼12.5% with Cucumis sativus seed oil; PSA/inflammation normalized
	Rat; testosterone–DMBA model	DMBA PAH with androgenic promotion; EBPS intervention	Ibrahim et al. (2022)	Directionally protective across doses	EBPS lowered PSA, suppressed 5α-R/Na^+^/K^+^-ATPase; decreased tumor mass
	Mouse xenograft; systemic BaP	Systemic BaP exposure	Zhang Z. et al. (2025)	Consistent BaP-dose effects on TME	Faster tumor growth; reduced intratumoral CD4^+^/CD8^+^ T-cell infiltration (immunosuppression)

Evidence strength (narrative): Strong—occupational long-term exposure; animal PAH, initiation/promotion. Moderate—traffic-related mixtures (including benzene) and high-temperature cooking proxies. Weak/inconsistent—residential proximity to emitters (ecological proxies). Adjusted estimates are shown where available; model specification and exposure error may influence precision.

### 3.1 Endocrine disruption via AhR–AR crosstalk and intracrine androgen rewiring

Endocrine disruption in this context refers to AH-associated disturbances of AR signaling and intracrine androgen biosynthesis (Li X. et al., 2024; Singh et al., 2024; Procházková et al., 2025). AHs, particularly PAHs such as BaP, together with dioxin-like aromatic ligands, perturb androgen signaling primarily through AhR, with potential relevance to both androgen-dependent and castration-resistant disease states (Chen X. et al., 2025). Upon ligand binding, AhR translocates to the nucleus and induces xenobiotic-metabolizing enzymes, including CYP1A1/1B1 (Grishanova and Perepechaeva, 2022; Szaefer et al., 2024). In parallel, AhR intersects the androgen receptor (AR) axis at several levels relevant to PCa biology, including AR stability, cofactor selection, and enhancer occupancy (Li X. et al., 2024). Mechanistic and translational studies suggest that PAH-activated AhR can suppress AR signaling, promote AR degradation, or, in a context-dependent manner, co-occupy androgen-response elements (AREs) with AR/aryl hydrocarbon receptor nuclear translocator (ARNT) to rewire hormonal programs; ligand type, dose, and disease stage may influence the direction of this effect (Zgarbová and Vrzal, 2022; Miller et al., 2023; Singh et al., 2024). Because AR signaling is heterogeneous in primary PCa and frequently altered in advanced disease, AH–AhR interference with AR programs may have stage-dependent consequences (Network, 2015; Robinson et al., 2015). In androgen-sensitive models, exogenous AhR activation often exerts anti-androgenic effects; in advanced or androgen-deprived settings, AhR signaling may become constitutively active and potentially sustain AR-target transcription, illustrating stage-dependent bidirectionality (Li S. et al., 2025).

**FIGURE 2:**
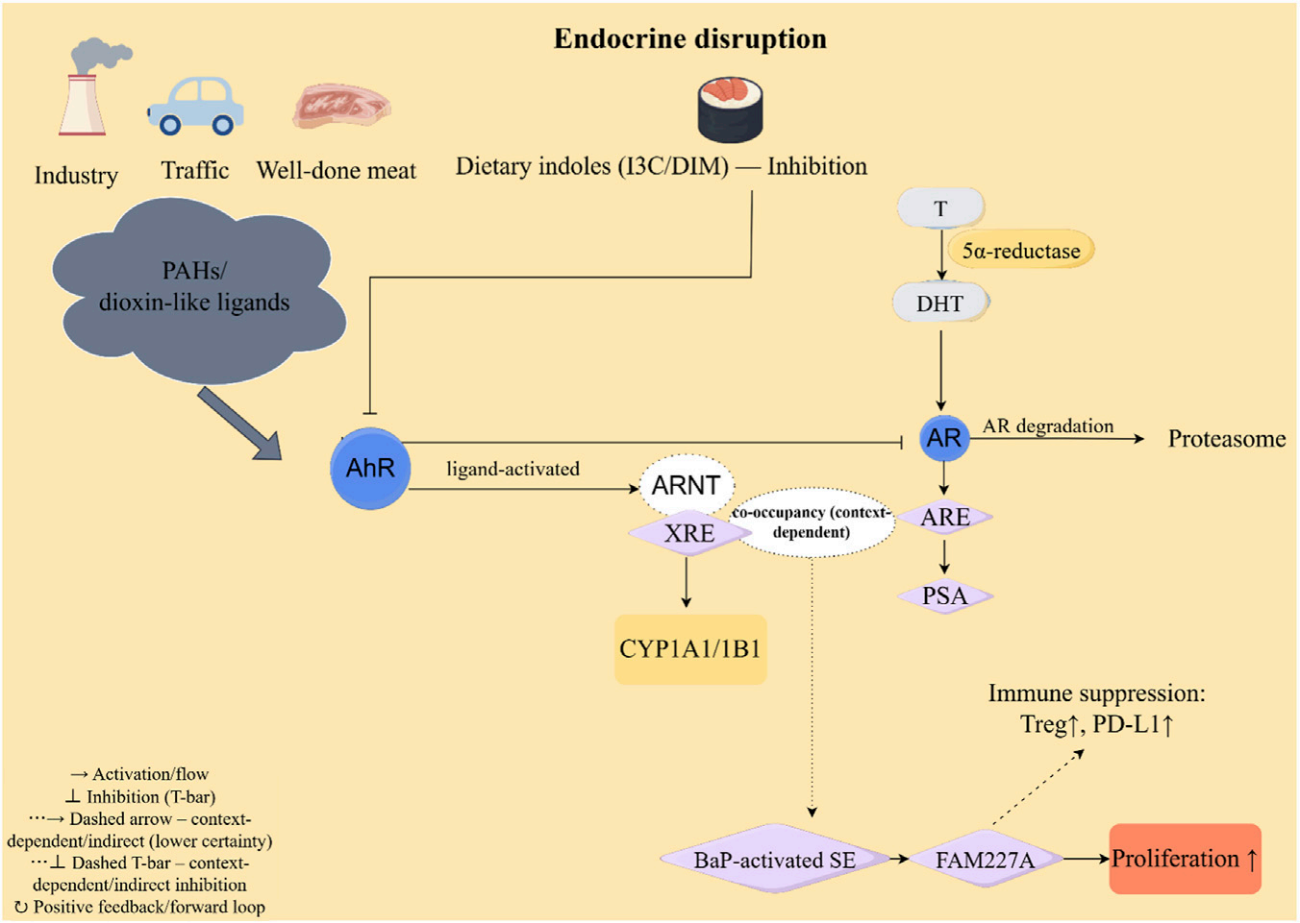
Endocrine disruption by aromatic hydrocarbons. PAHs and dioxin-like aromatic ligands activate AhR, induce CYP1A1/1B1, and interact with AR signaling in a context-dependent manner. This network links AR regulation, intracrine androgen metabolism, CYP1-mediated bioactivation, ROS/genotoxic stress, and BaP-responsive FAM227A activation. Diet-derived indoles may modulate AhR and antagonize AR signaling depending on ligand and dose context.

Classical endocrine-disrupting actions fall into two linked themes: direct interference with AR function and turnover, and disruption of intracrine androgen production, thereby reshaping the prostatic androgen milieu even under systemic androgen suppression (Zgarbová and Vrzal, 2022; Procházková et al., 2025). In LNCaP cells, experimental AhR ligands such as 3-methylcholanthrene suppress AR and accelerate proteasomal degradation; AhR-activating pollutants, including dioxin-like polychlorinated biphenyls (PCBs) and PAHs, can decrease prostate-specific antigen (PSA) output and inhibit 5α-R, the enzyme converting testosterone to dihydrotestosterone (DHT), consistent with attenuated AR signaling under selected environmental ligands (Singh et al., 2024; Procházková et al., 2025). Meanwhile, the prostate relies on intracrine conversion of adrenal precursors, including dehydroepiandrosterone (DHEA) and DHEA sulfate (DHEA-S), to active androgens across stromal and epithelial compartments; interference by aromatic pollutants with 3β-HSD, 17β-HSD, and 5α-R may modulate this local circuitry and provide a mechanistic route through which exposure could influence androgen-deprivation therapy (ADT) response (Zgarbová and Vrzal, 2022; Procházková et al., 2025). Exposure-responsive genetic susceptibility adds another layer: a BaP-responsive super-enhancer on chromosome 22 contains a common variant that strengthens an AhR motif and upregulates FAM227A upon BaP exposure, thereby promoting malignant behavior in prostate models (Singh et al., 2024). Importantly, not all AhR ligands exert equivalent effects; diet-derived indoles, including indole-3-carbinol (I3C) and 3,3′-diindolylmethane (DIM), can modulate AhR and antagonize AR signaling in preclinical contexts, underscoring ligand-, dose-, and disease-context dependence (Zgarbová and Vrzal, 2022; Tucci et al., 2023). As summarized in Figure 2, ligand-activated AhR links endocrine disruption to CYP1A1/1B1 induction and the ROS/genotoxic-stress axis discussed in Section 3.2.

### 3.2 Oxidative stress and DNA damage via AhR–CYP1 bioactivation and ROS/adduct formation

**FIGURE 3:**
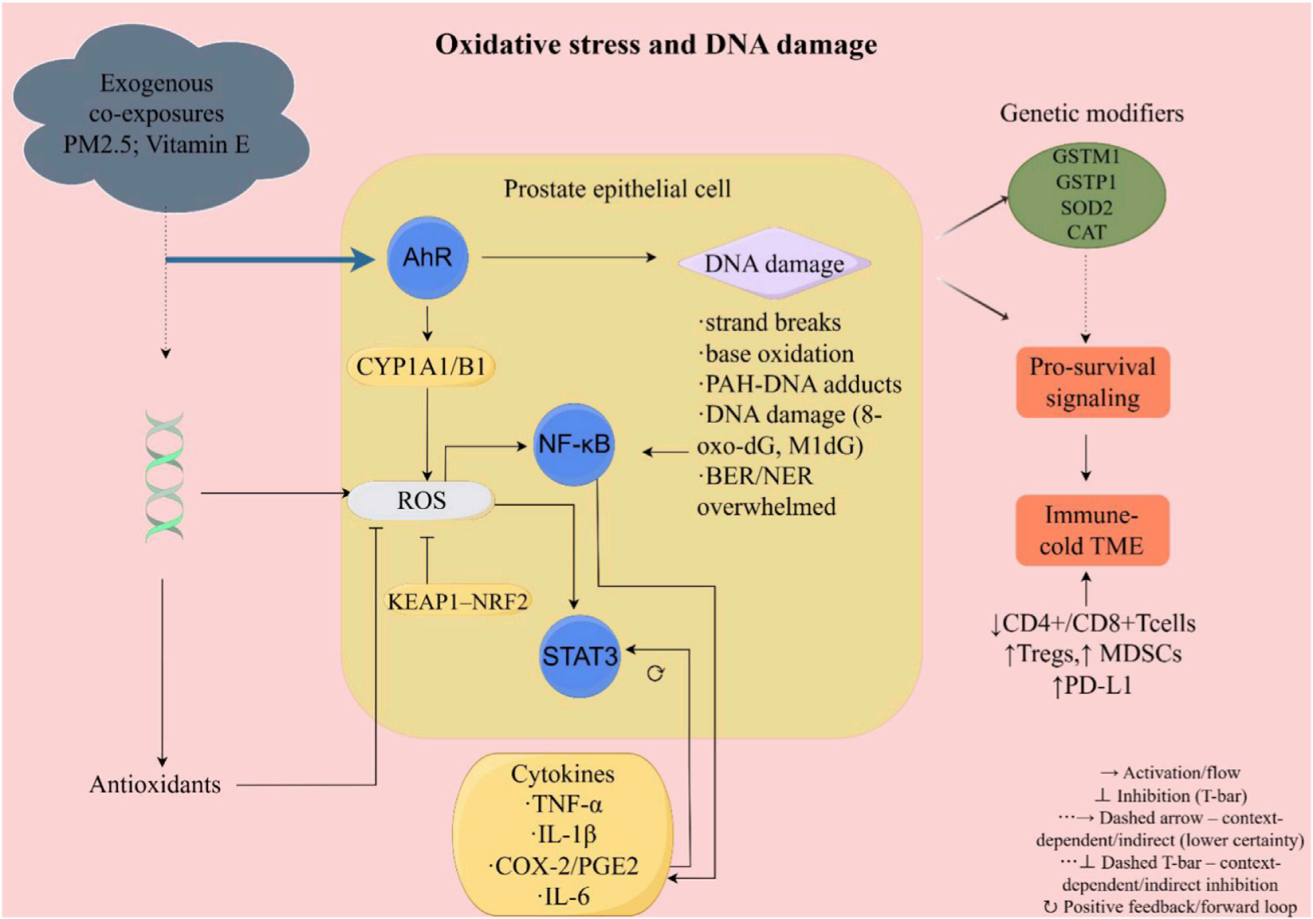
Oxidative stress and genotoxic injury downstream of AhR–CYP1 signaling. PAHs and co-occurring HCAs undergo AhR–CYP1A1/1B1-dependent bioactivation, increasing ROS generation, DNA damage, and DNA-adduct formation. ROS activates NF-κB and JAK2/STAT3 signaling, whereas KEAP1–NRF2 provides an adaptive antioxidant response. Host modifiers in detoxification, antioxidant defense, and DNA repair may influence susceptibility to AH-related prostate injury.

Building on Section 3.1, the AhR→CYP1 hand-off couples endocrine perturbation to redox and genotoxic stress. AHs, particularly PAHs, together with co-occurring cooking-derived HCAs, may promote PCa through a bioactivation-to-oxidative-injury-to-DNA-damage axis within the prostate, essentially an AhR→CYP1A1/1B1→ROS cascade that links metabolic activation to redox stress (Figure 3) (Bukowska et al., 2022; Liou et al., 2024; Mokhosoev et al., 2024). Human prostate tissue expresses CYP1 enzymes capable of activating PAHs and generating DNA-reactive intermediates *in situ*; PAH–DNA adducts, oxidative DNA lesions such as 8-oxo-2′-deoxyguanosine (8-oxo-dG), and lipid-peroxidation-derived M1dG adducts have been detected in prostate tumors, consistent with adduction-related genomic instability and oxidative injury (Guo et al., 2022; Hsieh et al., 2024; Maksymchuk et al., 2024). Mechanistically, BaP and related procarcinogens generate genotoxic electrophiles, including diol epoxides, while increasing ROS, thereby producing strand breaks, base oxidation, and lipid-peroxidation-derived lesions that may exceed base-excision and nucleotide-excision repair capacity (Bukowska et al., 2022; Liou et al., 2024). At the adaptive-response level, KEAP1 functions as a cysteine-rich redox sensor that restrains NRF2 under basal conditions but permits NRF2 stabilization and nuclear translocation under electrophilic or oxidative stress. Nuclear NRF2 binds antioxidant-response elements (AREs) and induces cytoprotective and phase-II detoxification genes, including NQO1, HMOX1/HO-1, GCLC/GCLM, GST-family enzymes, SOD2, CAT, and GPX1. In PCa, this pathway is context dependent: transient NRF2 activation may buffer PAH-induced ROS and electrophilic injury, whereas persistent NRF2–HO-1/NQO1 activity may support redox adaptation, tumor-cell survival, and therapy resistance in stressed malignant cells (Xu et al., 2013; Jayasooriya et al., 2015; Li et al., 2021; Tossetta et al., 2023b). Dietary bioactives provide a useful mechanistic contrast to pollutant-driven oxidative injury. In PCa, NRF2 antioxidant signaling intersects with NF-κB-driven inflammation and AR signaling, providing a mechanistic basis for considering dietary modulators of redox and inflammatory tone (Khurana and Sikka, 2018). Green-tea catechins, particularly EGCG, have been linked to reduced inflammatory signaling and systemic oxidative-stress biomarkers in men with prostate cancer before prostatectomy (Henning et al., 2015). In xenograft models, brewed green-tea polyphenols localized to prostate tumors and were associated with reduced oxidative damage and angiogenesis-related signaling (Henning et al., 2012). Cruciferous-vegetable-derived indoles, including I3C and DIM, can regulate phase I/II detoxification enzymes, Akt/NF-κB signaling, cell-cycle control, and apoptosis in prostate cancer models (Sarkar and Li, 2004). Consistently, DIM inhibited prostate carcinogenesis in the TRAMP model and modulated proliferation, apoptosis, and cell-cycle regulators (Cho et al., 2011). Together, these findings suggest that dietary factors may influence PCa biology less as stand-alone preventive agents than as modulators of redox, inflammatory, hormonal, and genotoxic-stress pathways that intersect with AH-induced oxidative injury. In PC-3 cells, BaP increases proliferation, DNA breaks, and mutagenesis; these effects are attenuated by CYP1/AhR blockade or JAK2 inhibition, linking the AhR→CYP1A1/1B1 axis to JAK2–STAT3 signaling and pro-survival transcriptional programs (Gao et al., 2020). Complementary data show that human CYP1B1 can convert BaP-7,8-dihydrodiol and selected HCAs into potent genotoxic metabolites and elevate chromosomal translocations, supporting a bioactivation-dependent hazard gradient (Bukowska et al., 2022; Mokhosoev et al., 2024; Montano et al., 2025).

Biomarker studies connect real-world exposure to molecular injury and, in some cases, to clinical phenotype. In surgical cohorts, hair dosimetry for 2-amino-1-methyl-6-phenylimidazo[4,5-b]pyridine (PhIP), a long-term marker of cooking-derived HCA exposure, has been associated with higher PSA and worse Gleason grade (Guo et al., 2022). Oxidative and lipid-peroxidation footprints are common: acrolein-derived 1,N^2^-propano-dG appears in roughly half of prostate specimens, and lipid-peroxidation adducts are enriched in higher-grade disease (Guo et al., 2022; Biesiadecki et al., 2024). Host variation in detoxification and repair pathways—glutathione S-transferase mu 1 (GSTM1), glutathione S-transferase pi 1 (GSTP1), superoxide dismutase 2 (SOD2), catalase (CAT), glutathione peroxidase 1 (GPX1), and X-ray repair cross-complementing 1 (XRCC1), among others—modulates susceptibility by shaping internal dose, adduct burden, and the tally of unresolved lesions (Álvarez-González et al., 2024; van Campen et al., 2025). PAH-carrying particulate mixtures, such as PM2.5, may further intensify oxidative damage in prostate tissue, consistent with traffic-related co-exposure (Lee et al., 2024). Notably, co-exposures or metabolic modifiers may amplify this axis: vitamin E (α-tocopherol), in specific experimental contexts, has been reported to upregulate PAH-activating CYPs in prostate epithelium, elevate ROS, and enhance BaP-induced transformation (Jiang, 2024; Zhu et al., 2024). Preclinical intervention studies provide mechanistic support for this redox axis. In a testosterone–BaP rat model, oxidative stress increased in parallel with tumor incidence, whereas Cucumis sativus seed oil restored antioxidant defenses, lowered inflammatory cytokines, and reduced tumor incidence from 75% to 12.5%, suggesting that redox-targeted mitigation is experimentally feasible (Bakam et al., 2023). Sustained oxidative stress does more than injure macromolecules; it also conditions the epigenome and stabilizes exposure-linked transcriptional programs, thereby motivating the next section on epigenetics (Wang et al., 2023).

### 3.3 Epigenetic remodeling and exposure-linked transcriptional memory via DNMT1/HDAC6 and super-enhancers

Following oxidative and genotoxic stress, four exposure-responsive epigenetic layers merit particular attention: DNA methylation maintenance, histone acetylation/deacetylation dynamics, AhR-directed enhancer remodeling, and ncRNA-mediated regulation. DNA methylation maintenance. The prioritization of DNA methyltransferase 1 (DNMT1) among air-pollution–PCa core genes suggests that aberrant maintenance methylation may participate in exposure-related tumorigenesis. BaP exposure has also been linked to altered DNMT activity and promoter methylation of tumor-suppressor loci, including canonical PCa targets such as glutathione S-transferase P1 (GSTP1) (Wang et al., 2023; Li Y. et al., 2025). Histone acetylation/deacetylation dynamics. HDAC6 has emerged from network-based analyses as a candidate node linking stress and inflammatory signaling to chromatin remodeling, cytoskeletal dynamics, and AR trafficking (Huang et al., 2024; Li Y. et al., 2025). This interpretation is supported indirectly by integrative epigenetic taxonomy studies showing that primary prostate cancer comprises distinct AR/chromatin states with subtype-specific transcriptional control, highlighting the broader biological relevance of chromatin remodeling in prostate cancer progression (Stelloo et al., 2018).

AhR-directed super-enhancer remodeling. A more explicit environment–genome–epigenome link has been reported for BaP: a BaP-responsive super-enhancer at 22q harbors a functional variant that strengthens an AhR motif, augments AhR binding, and increases FAM227A expression upon exposure, thereby promoting malignant phenotypes in PCa models and suggesting exposure-responsive enhancer activity (Fan et al., 2024). Transcription-factor crosstalk. AhR, after ligand activation and dimerization with ARNT at xenobiotic response elements (XREs), can interface with androgen receptor (AR) signaling and potentially reshape enhancer occupancy relevant to progression and resistance (Singh et al., 2024; Procházková et al., 2025). Notably, BaP-activated AhR–AR crosstalk may recruit or cooperate with epigenetic silencers such as DNMT1 and HDAC1 at chromatin, providing a mechanistic link between endocrine perturbation and focal transcriptional repression (Farooqi et al., 2023; Singh et al., 2024). ncRNA-mediated regulation provides an additional layer of control. Enrichment analyses highlighting “microRNAs in cancer” are consistent with the possibility that AH-perturbed microRNA networks may modulate AR signaling, cell-cycle control, and stress-response pathways (Li et al., 2022; Gan et al., 2024; Krajka-Kuźniak et al., 2024). Exposure and metabolic context. Urinary BaP and related organic pollutants have been associated with increased PCa risk; polymorphisms in xenobiotic-metabolism pathways, including CYP1A1 and GSTP1 variants, may influence intracellular dose and downstream regulatory responses (Medjani et al., 2020; Álvarez-González et al., 2023; Goertzen et al., 2024).

Taken together, AH exposure may reshape the prostate cancer epigenome through AhR-centered enhancer activity, DNMT1/HDAC6-associated chromatin regulation, and ncRNA-mediated stress responses. These changes can converge on AR-dependent transcription, survival signaling, cell-cycle regulation, and EMT, and may persist beyond the initiating exposure as relatively stable regulatory states.

### 3.4 Altered proliferation, apoptosis, and EMT via JAK2/STAT3 signaling

Beyond epigenetic remodeling, AHs such as BaP can reshape PCa cell behavior by promoting proliferation, limiting apoptosis, enhancing migration, and potentially engaging vascular-remodeling programs, primarily through JAK2/STAT3 and cooperating circuits that integrate oxidative and inflammatory cues (Gao et al., 2020; Sadrkhanloo et al., 2023). In PC-3 cells, BaP increases viability and S-phase entry and upregulates Cyclin D1 and CDK4; pharmacologic JAK2 blockade with AG490 or AhR antagonism with CH223191 attenuates these effects, supporting involvement of the AhR–JAK2/STAT3 axis. Concurrently, BaP induces DNA strand breaks yet can dampen apoptosis by shifting key regulators, including reduced Bax and increased Bcl-2, while limiting p53/FOXO1-mediated death signals. BaP has also been reported to elevate MDM2 and suppress FOXO1, thereby reducing apoptosis and increasing clonogenic survival *in vitro* (Gao et al., 2020; Shahid et al., 2023; Zhang Z. et al., 2025). Migratory and invasive features also increase: BaP enhances transwell migration, elevates EMT-associated transcription factors such as Snail and Slug as well as matrix metalloproteinase-9 (MMP-9), and lowers E-cadherin. These phenotypes appear to depend, at least in part, on JAK2/STAT3 signaling and are attenuated by pathway inhibition (Gao et al., 2020; Sadrkhanloo et al., 2023). Although direct evidence linking AH exposure to angiogenesis or vascular remodeling in prostate cancer remains limited, AH-responsive oxidative, inflammatory, and JAK2/STAT3-associated programs may provide a plausible upstream context for vascular-related changes. In prostate adenocarcinoma, VEGF-A immunoexpression has been associated with higher PSA levels, higher tumor grade, and more advanced lesions, supporting the biological relevance of angiogenic signaling in aggressive PCa. Therefore, vascular remodeling should currently be interpreted as a potential downstream consequence of AH-induced redox and inflammatory signaling rather than as a firmly established AH-specific mechanism in prostate cancer (Pănuş et al., 2021). Taken together, these tumor-cell-intrinsic changes may be amplified by inflammatory circuits, linking AH exposure to pro-survival, pro-migratory, and potentially therapy-resistant phenotypes under chronic exposure.

### 3.5 Inflammatory signaling and immunosuppressive TME remodeling via kynurenine–AhR and COX-2/PGE2

**FIGURE 4:**
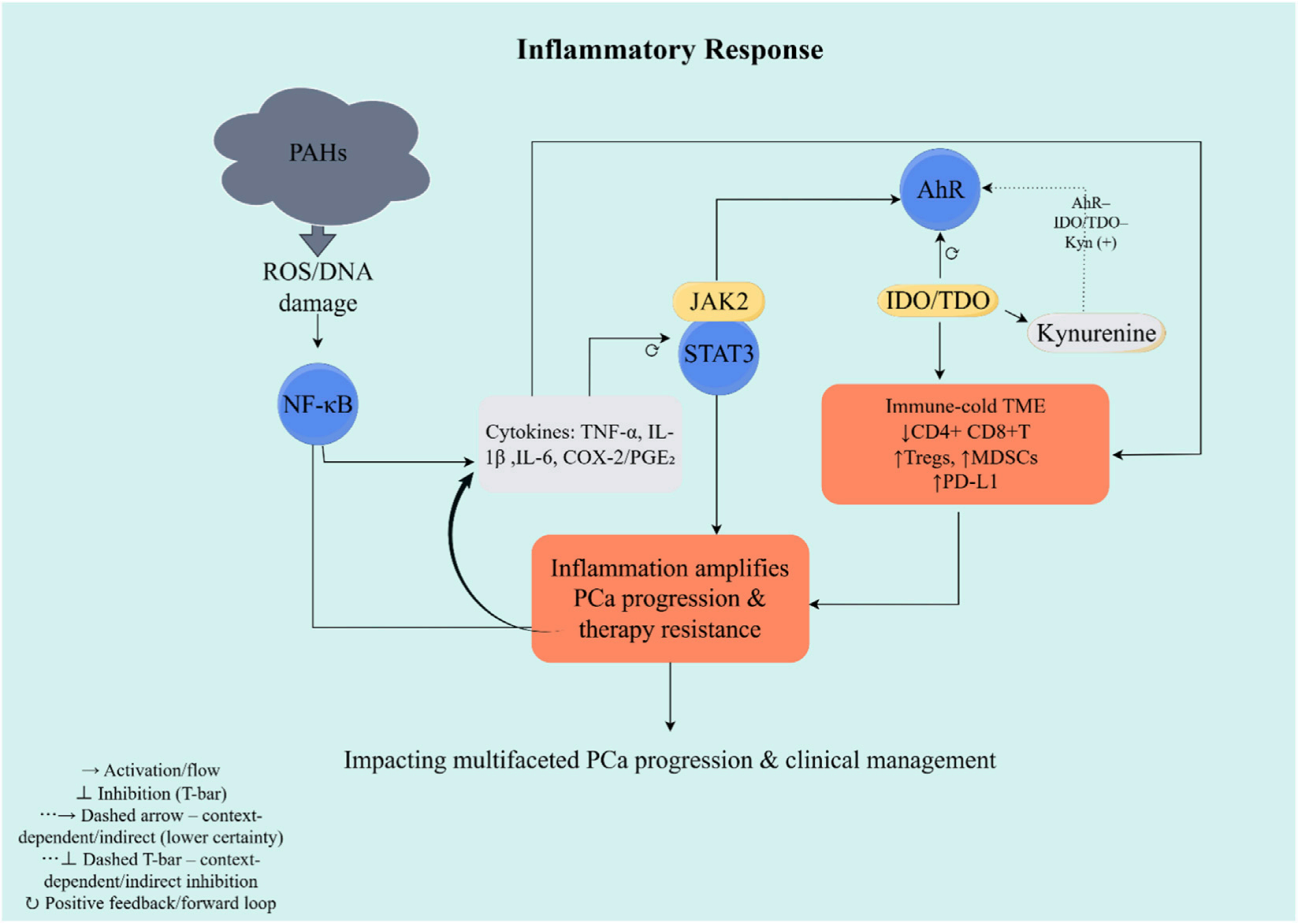
Inflammatory and immune remodeling under PAH exposure. PAH-induced ROS and DNA damage activate NF-κB and JAK2/STAT3 signaling, promoting inflammatory cytokines and COX-2/PGE2 activity. The IDO/TDO–kynurenine–AhR axis may further contribute to an immunosuppressive or immune-cold tumor microenvironment, linking chronic inflammation to prostate cancer progression and therapy resistance.

Extending these cell-intrinsic programs to the tumor microenvironment, exposure to AHs such as BaP can trigger inflammatory cascades that support PCa progression across epithelial and stromal compartments (Liu et al., 2024; Zhang Z. et al., 2025). ROS and DNA damage can activate nuclear factor-κB (NF-κB) and JAK/STAT signaling, elevating tumor necrosis factor-α (TNF-α), interleukin-1β (IL-1β), interleukin-6 (IL-6), and cyclo-oxygenase-2/prostaglandin E2 (COX-2/PGE2), thereby creating a cytokine milieu that sustains proliferation and survival (Cuenca-Escalona et al., 2024; Guo et al., 2024; Hu et al., 2024). IL-6, in particular, can reinforce STAT3-dependent signaling, thereby closing the loop with the JAK2/STAT3 proliferative axis discussed in Section 3.4 (Sadrkhanloo et al., 2023; Hu et al., 2024). With chronic BaP exposure, animal models show accelerated tumor growth and reduced intratumoral CD4^+^ and CD8^+^ T-cell infiltration. Together with broader evidence implicating Tregs, MDSCs, and PD-L1 in immunosuppressive PCa microenvironments, these findings support a shift toward an immune-cold or immune-suppressed TME that favors growth, invasion, and metastasis (Taş et al., 2024; Zhang Z. et al., 2025). These features may align with activation of the AhR–indoleamine-2,3-dioxygenase/tryptophan-2,3-dioxygenase (IDO/TDO)–kynurenine pathway and with COX-2/PGE2 circuits that can skew myeloid and lymphoid composition (Cuenca-Escalona et al., 2024; Li S. et al., 2025). Notably, antioxidant or anti-inflammatory interventions, including COX-2 or JAK/STAT3 targeting, can mitigate related inflammatory and survival outputs in preclinical settings, suggesting that multipronged strategies combining redox control with checkpoint and/or STAT3 blockade warrant further evaluation rather than relying on single-node approaches (Gao et al., 2020; Cuenca-Escalona et al., 2024; Kassab, 2025). Inflammation-driven survival signals converge with endocrine, genotoxic, and epigenetic programs at shared hubs, including JAK2/STAT3, NF-κB, PI3K/AKT, and MAPK, building a resilient crosstalk network that may promote therapy resistance (Guo et al., 2024; Hu et al., 2024; Liu et al., 2024). Accordingly, exposure-informed and pathway-layered interventions may offer advantages over narrowly focused approaches, although direct clinical evidence remains limited. These interacting inflammatory and immune-remodeling pathways are summarized in Figure 4.

## 4 Exposure-reduction strategies informed by AH–PCa mechanisms

**FIGURE 5:**
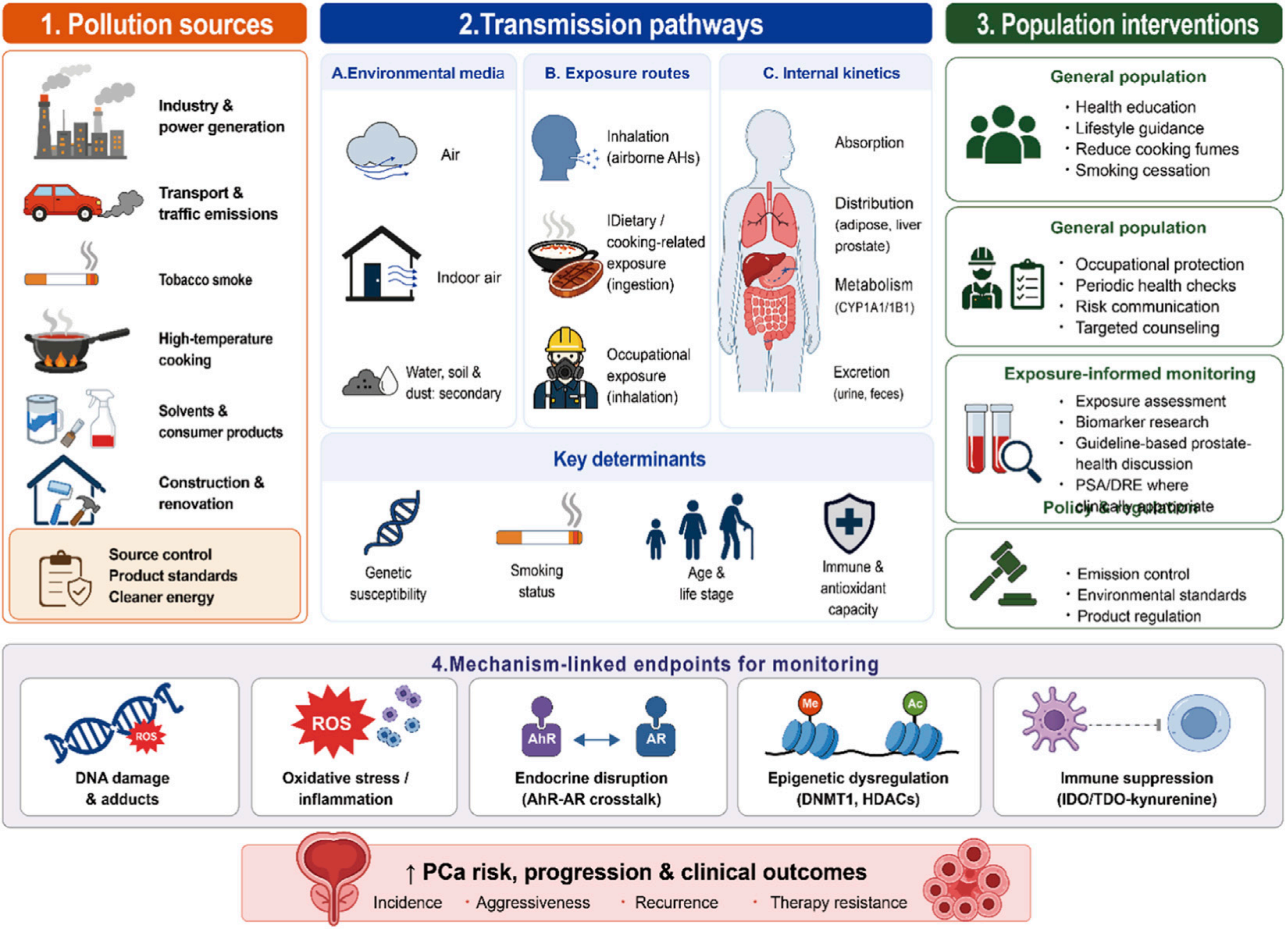
Mechanism-informed framework for aromatic hydrocarbon exposure reduction and monitoring. This framework links major AH/PAH pollution sources with key transmission pathways, including air, indoor air, inhalation, cooking-related dietary exposure, and occupational exposure. Population interventions include general exposure reduction, protection of high-risk or susceptible groups, exposure-informed monitoring, and policy regulation. Mechanism-linked endpoints for monitoring include DNA damage and adducts, oxidative stress and inflammation, AhR–AR crosstalk, epigenetic dysregulation, and immune suppression, which may relate to PCa risk, progression, and clinical outcomes.

This section translates the mechanistic framework described above into exposure-reduction principles relevant to population-level prevention and clinical risk communication. Rather than serving as a stand-alone public-health review, it focuses on measures most directly aligned with AH–PCa mechanisms: lowering benzene and PAH inputs, reducing cumulative biological dose, and identifying populations with sustained or high-intensity exposure. Because AHs arise from industrial emissions, transportation, tobacco smoke, indoor solvents, and high-temperature cooking, this framework is organized around source control, pathway-specific mitigation, and exposure-informed protection of susceptible populations (Figure 5). These strategies should therefore be interpreted as mechanism-informed exposure-reduction approaches rather than as evidence-proven PCa-specific prevention interventions.

### 4.1 Source control to reduce upstream AH burden

Source control is the most upstream strategy for reducing biologically relevant AH exposure burden because it lowers the external inputs that feed AhR activation, CYP1A1/1B1-mediated bioactivation, oxidative stress, and DNA-adduct formation (Goldberg et al., 2022; Chen Y. et al., 2025). Regulatory and industrial emission-control approaches that reduce BTX, especially benzene, and PAH concentrations may therefore lower upstream exposure inputs relevant to AH-linked biological pathways (United States Environmental Protection Agency, 2014; United States Environmental Protection Agency, 2015; Adeyeye et al., 2025). Cleaner combustion, improved emission capture, and safer substitution in high-emission industries may further reduce PAH-rich mixtures, particularly in petroleum, chemical, manufacturing, and waste-related settings (Badami et al., 2023; United States Environmental Protection Agency, 2023; Wei et al., 2025). Product-level controls on solvent-containing materials may also reduce indoor AH exposure, particularly in poorly ventilated environments (Bergengren et al., 2023; ECHA, 2025b; ECHA, 2025a).

### 4.2 Pathway-specific mitigation across major exposure routes

Across inhalational, dietary, indoor, and occupational routes, mitigation is most relevant when it reduces cumulative biological dose rather than exposure category alone (Jiang et al., 2024; Teixeira et al., 2025a). Ambient mitigation is most relevant to AH–PCa biology when it reduces traffic- and industry-derived benzene, BTX, and PAH mixtures that contribute to chronic inhalational exposure (Wu et al., 2021; 2023; Mogashane et al., 2024; Cerón Bretón et al., 2025). Indoor and dietary sources are relevant to clinical risk communication because tobacco smoke, solvent-containing products, and high-temperature cooking can provide repeated low-level inputs into PAH/HCA-linked oxidative and genotoxic pathways (Jiang et al., 2024; Chamseddine et al., 2025; Simona et al., 2025). Reducing tobacco smoke, unnecessary solvent exposure, and high-temperature cooking emissions may lower inputs into oxidative, genotoxic, and inflammatory pathways relevant to PCa biology (Harding-Smith et al., 2024; Wu et al., 2024).

Occupational exposure remains important because firefighting, vehicle repair, manufacturing, printing, and related jobs can involve repeated or high-intensity VOC and PAH contact (Lothrop et al., 2025; Teixeira et al., 2025a). Workplace protection, exposure-duration control, and occupational-health follow-up may reduce cumulative exposure and provide a practical setting for exposure documentation (Chakr and Sav, 2024).

### 4.3 Exposure-informed protection of susceptible populations

For populations with sustained or high-intensity AH exposure, protection should combine risk communication, exposure documentation, feasible exposure reduction, and guideline-based prostate-health discussion. These domains should be framed as supportive risk-management tools rather than as AH-specific screening recommendations (Goldberg et al., 2022; Youogo et al., 2022). In occupational-health or clinical settings, men with substantial exposure histories may benefit from guideline-based discussion of prostate-health assessment, including PSA testing and DRE where clinically appropriate, although exposure alone should not determine screening decisions (Chakr and Sav, 2024; Cornford et al., 2024; Xu et al., 2024). For residents near major benzene- or PAH-emitting facilities, exposure assessment and risk communication may be more appropriate than broad AH-specific screening recommendations.

Education should emphasize major AH sources, exposure routes, and feasible reduction behaviors (Health Canada, 2025). For high-exposure workers, correct PPE use and exposure-time reduction remain practical measures, while smoking cessation and reduced high-temperature cooking may lower relevant co-exposures (Bergengren et al., 2023; Chakr and Sav, 2024; Matthaios et al., 2024; Wan et al., 2024). Because PCa has a long latency, long-term occupational-health follow-up may be useful for exposure documentation and risk communication in high-exposure groups (Bergengren et al., 2023). In research or sentinel cohorts, integrating exposure biomarkers with susceptibility markers may help clarify inter-individual vulnerability (Álvarez-González et al., 2024). Longitudinal studies should link personal exposure monitoring, biomarkers of internal dose or DNA damage, and PCa-relevant outcomes (Goldberg et al., 2022; Khoshakhlagh et al., 2025). This framework links exposure reduction with molecular endpoints such as internal dose, DNA damage, oxidative stress, and immune remodeling, and provides the exposure context for Section 5 without treating AH exposure as an independent determinant of PCa management.

## 5 Exposure-informed clinical considerations for PCa management

Building on the mechanism-informed exposure-reduction framework outlined in Section 4, this section considers how AH exposure information may inform patient-level PCa care without overextending current evidence. AH exposure should not be used as an independent determinant of screening or treatment selection; however, structured exposure assessment may provide context for risk discussion, exposure-reduction counseling, and hypothesis generation regarding progression or treatment resistance. Three clinically relevant domains are considered: structured exposure history, exposure-related mechanisms of prognosis and resistance, and guideline-based follow-up plus patient education.

### 5.1 Structured exposure history for risk assessment and diagnostic context

AHs, including PAHs and MAHs, are increasingly implicated in PCa development, but exposure history should be viewed as contextual information rather than as a stand-alone diagnostic tool (Bergengren et al., 2023; Álvarez-González et al., 2024). In clinical or occupational-health settings, structured exposure assessment may support risk discussion and feasible exposure-reduction counseling, particularly for individuals with sustained occupational exposure (Lewis-Mikhael et al., 2016; Bijoux et al., 2022; Barul et al., 2025).

Relevant exposure-history domains include occupation or work process and duration, residential proximity to traffic or industrial sources, indoor exposures such as tobacco smoke or solvents, high-temperature cooking practices, major combustion events such as fires or burn pits, and PPE use (Choi et al., 2022; Zapata-Marin et al., 2022; Adeyeye et al., 2025). When available, exposure biomarkers may complement questionnaires, but incomplete occupational records, residential mobility, and limited monitoring data mean that exposure-informed assessment should remain a scalable framework rather than a rigid clinical requirement.

### 5.2 Exposure-related mechanisms as prognostic and treatment-resistance modifiers

PAHs, particularly BaP, may influence pathways related to PCa progression and treatment resistance, although direct clinical evidence remains limited (Liou et al., 2024; Fang et al., 2025; Wang et al., 2025). Mechanistically, exposure-related AhR–AR crosstalk, oxidative and genotoxic stress, and inflammatory or immune-microenvironment remodeling may intersect with established resistance pathways (Fang et al., 2025). Because AR reprogramming and AR splice variants are central to CRPC biology, PAH-related AhR–AR signaling provides a plausible but insufficiently validated link between exposure and resistance-associated phenotypes (Robinson et al., 2015; Han et al., 2024; Singh et al., 2024). In parallel, BaP exposure has been reported to reduce cytotoxic T-cell infiltration and promote immunosuppressive remodeling, whereas oxidative stress and JAK2/STAT3-linked inflammation may create a microenvironment associated with aggressiveness and reduced treatment sensitivity (Sadrkhanloo et al., 2023; Liou et al., 2024; Zhang Z. et al., 2025). Thus, AH exposure history may complement mechanistic interpretation and resistance-risk hypothesis generation, but it should not replace established molecular or clinical prognostic frameworks (Li J. et al., 2024; Álvarez-González et al., 2024; Chen Y. et al., 2025).

### 5.3 Exposure-informed surveillance, counseling, and longitudinal follow-up

Exposure information may complement personalized PCa care, but it should not independently determine screening intervals, follow-up intensity, or oncologic treatment selection. This view aligns with precision environmental health, which integrates environmental, molecular, and clinical information to support prevention-oriented counseling and risk contextualization (Baccarelli et al., 2023).

For individuals with substantial or prolonged PAH/MAH exposure, follow-up discussion should remain guideline-based and individualized (Gao et al., 2022; Ledda et al., 2023; Bijoux et al., 2025). PSA testing and DRE should remain anchored in existing clinical guidelines and shared decision-making, whereas adjunct exposure biomarkers, such as urinary VOCs, hair-based carcinogen measures, or DNA-adduct proxies, remain investigational tools for assessing internal dose or genotoxic injury in occupational-health or research contexts (Guo et al., 2022; Wei et al., 2023; Dawson et al., 2024; Turesky et al., 2025). Practical counseling should focus on feasible exposure reduction, including PPE use, exposure-time reduction, and avoidance of unnecessary combustion or solvent exposure (Chakr and Sav, 2024; Simona et al., 2025).

Accordingly, exposure-informed follow-up should focus on exposure documentation, feasible risk reduction, guideline-based prostate-health discussion, and long-term linkage of exposure metrics with PCa-relevant molecular and clinical outcomes (Gao et al., 2022).

### 5.4 Patient education and feasible lifestyle counseling

Patient education remains a practical component of exposure-informed care when framed as risk communication rather than treatment modification. Counseling can explain how AHs, including BaP and other PAHs encountered through air, tobacco smoke, occupational combustion, solvents, and high-temperature cooking, may intersect with oxidative, endocrine, genotoxic, and immune pathways relevant to PCa biology (Bukowska et al., 2022; Zhao et al., 2023; Singh et al., 2024; Zhang Z. et al., 2025). Feasible counseling can emphasize smoking cessation, reduced high-temperature cooking emissions, avoidance of unnecessary solvent or combustion exposure, and dietary patterns that reduce mutagen-forming cooking practices while supporting redox and inflammatory balance (Tan and Norhaizan, 2021; Xing et al., 2023; Lin et al., 2025).

For high-exposure workers, counseling may also include correct PPE use and exposure-time reduction (Andersen and Saber, 2025; Teixeira et al., 2025b). Overall, patient education, feasible exposure reduction, and guideline-based follow-up can help patients recognize environmental contributors to PCa biology while maintaining realistic, evidence-based care.

## 6 Conclusions and future perspectives

Current evidence supports a biologically plausible link between environmental and occupational AH exposure and PCa, although causal inference remains constrained by exposure misclassification, mixture complexity, residual confounding, and limited longitudinal biomarker data. Epidemiological studies, occupational cohorts, and experimental models collectively suggest that prolonged AH exposure may contribute to PCa risk and progression through intersecting mechanisms, including endocrine disruption, AhR–CYP1-mediated oxidative and genotoxic injury, DNA-adduct formation, epigenetic reprogramming, inflammatory signaling, immune-microenvironment remodeling, and treatment-resistance-associated pathways.

These findings support a mechanism-informed framework in which exposure reduction, structured exposure history, and patient education are viewed as complementary tools for risk discussion and guideline-based follow-up rather than as stand-alone clinical decision rules. Future research should prioritize standardized exposure assessment, improved reconstruction of lifetime AH exposure, integration of biomarkers such as DNA adducts and oxidative-stress markers into prospective cohorts, and prostate-specific experimental systems that connect AhR, NRF2, AR signaling, inflammation, immune remodeling, and therapy resistance. Such efforts will be essential for clarifying causality, identifying susceptible populations, and translating environmental carcinogenesis mechanisms into realistic prevention and clinical-support strategies.

## Glossary

AHs: Aromatic hydrocarbons
MAHs: Monocyclic aromatic hydrocarbons
PAHs: Polycyclic aromatic hydrocarbons
BTX: Benzene–toluene–xylene
VOCs: Volatile organic compounds
PM2.5: Fine particulate matter ≤2.5 μm
BaP: Benzo[a]pyrene
DMBA: 7,12-Dimethylbenz[a]anthracene
HCAs: Heterocyclic amines
PhIP: 2-Amino-1-methyl-6-phenylimidazo[4,5-b]pyridine
PCa: Prostate cancer
CRPC: Castration-resistant prostate cancer
TME: Tumor microenvironment
EMT: Epithelial–mesenchymal transition
PSA: Prostate-specific antigen
DRE: Digital rectal examination
ADT: Androgen-deprivation therapy
AhR: Aryl hydrocarbon receptor
ARNT: Aryl hydrocarbon receptor nuclear translocator
AR: Androgen receptor
ARE: Androgen-response element
NF-κB: Nuclear factor-κB
STAT3: Signal transducer and activator of transcription 3
KEAP1: Kelch-like ECH–associated protein 1
NRF2: Nuclear factor erythroid 2–related factor 2
XREs: Aryl hydrocarbon response elements (xenobiotic)
AR-V7: Androgen receptor variant 7
JAK/STAT: Janus kinase/signal transducer and activator of transcription
PI3K/AKT: Phosphoinositide 3-kinase/protein kinase B
MAPK: Mitogen-activated protein kinase
5α-R: 5α-reductase
CYP1A1/1B1: Cytochrome P450 1A1/1B1
COX-2: Cyclo-oxygenase-2
PGE2: Prostaglandin E2
IDO/TDO: Indoleamine 2,3-dioxygenase/tryptophan 2,3-dioxygenase
ROS: Reactive oxygen species
M1dG: Lipid peroxidation–derived malondialdehyde–dG adduct
8-oxo-dG: 8-oxo-2′-deoxyguanosine
DNMT1: DNA methyltransferase 1
HDAC6: Histone deacetylase 6
FAM227A: FAM227A (Family with sequence similarity 227 member A)
XRCC1: X-ray repair cross-complementing 1
SOD2: Superoxide dismutase 2
CAT: Catalase
GPX1: Glutathione peroxidase 1
GSTM1: Glutathione S-transferase mu 1
GSTP1: Glutathione S-transferase pi 1
ncRNAs: Non-coding RNAs
PD-L1: Programmed death-ligand 1
Tregs: Regulatory T cells
MDSCs: Myeloid-derived suppressor cells
DHEA: Dehydroepiandrosterone
DHT: Dihydrotestosterone
PPE: Personal protective equipment.
